# Steering the course of CAR T cell therapy with lipid nanoparticles

**DOI:** 10.1186/s12951-024-02630-1

**Published:** 2024-06-28

**Authors:** Muhammad Babar Khawar, Ali Afzal, Yue Si, Haibo Sun

**Affiliations:** 1 Institute of Translational Medicine, Medical College, Yangzhou University, Yangzhou, China; 2 Jiangsu Key Laboratory of Experimental & Translational Non-Coding RNA Research Yangzhou, Yangzhou, China; 3 Applied Molecular Biology and Biomedicine Lab, Department of Zoology, University of Narowal, Narowal, Pakistan; 4 Shenzhen Institute of Advanced Technology, University of Chinese Academy of Sciences, Shenzhen, 518055, Guangdong China; 5 Molecular Medicine and Cancer Therapeutics Lab, Department of Zoology, Faculty of Sciences and Technology, University of Central Punjab, Lahore, Pakistan

**Keywords:** Chimeric antigen receptor, Lipid nanoparticles, Immunotherapy, mRNA delivery, Nonviral transduction

## Abstract

Lipid nanoparticles (LNPs) have proven themselves as transformative actors in chimeric antigen receptor (CAR) T cell therapy, surpassing traditional methods and addressing challenges like immunogenicity, reduced toxicity, and improved safety. Promising preclinical results signal a shift toward safer and more effective CAR T cell treatments. Ongoing research aims to validate these findings in clinical trials, marking a new era guided by LNPs utility in CAR therapy. Herein, we explore the preference for LNPs over traditional methods, highlighting the versatility of LNPs and their effective delivery of nucleic acids. Additionally, we address key challenges in clinical considerations, heralding a new era in CAR T cell therapy.

**Graphical Abstract:**

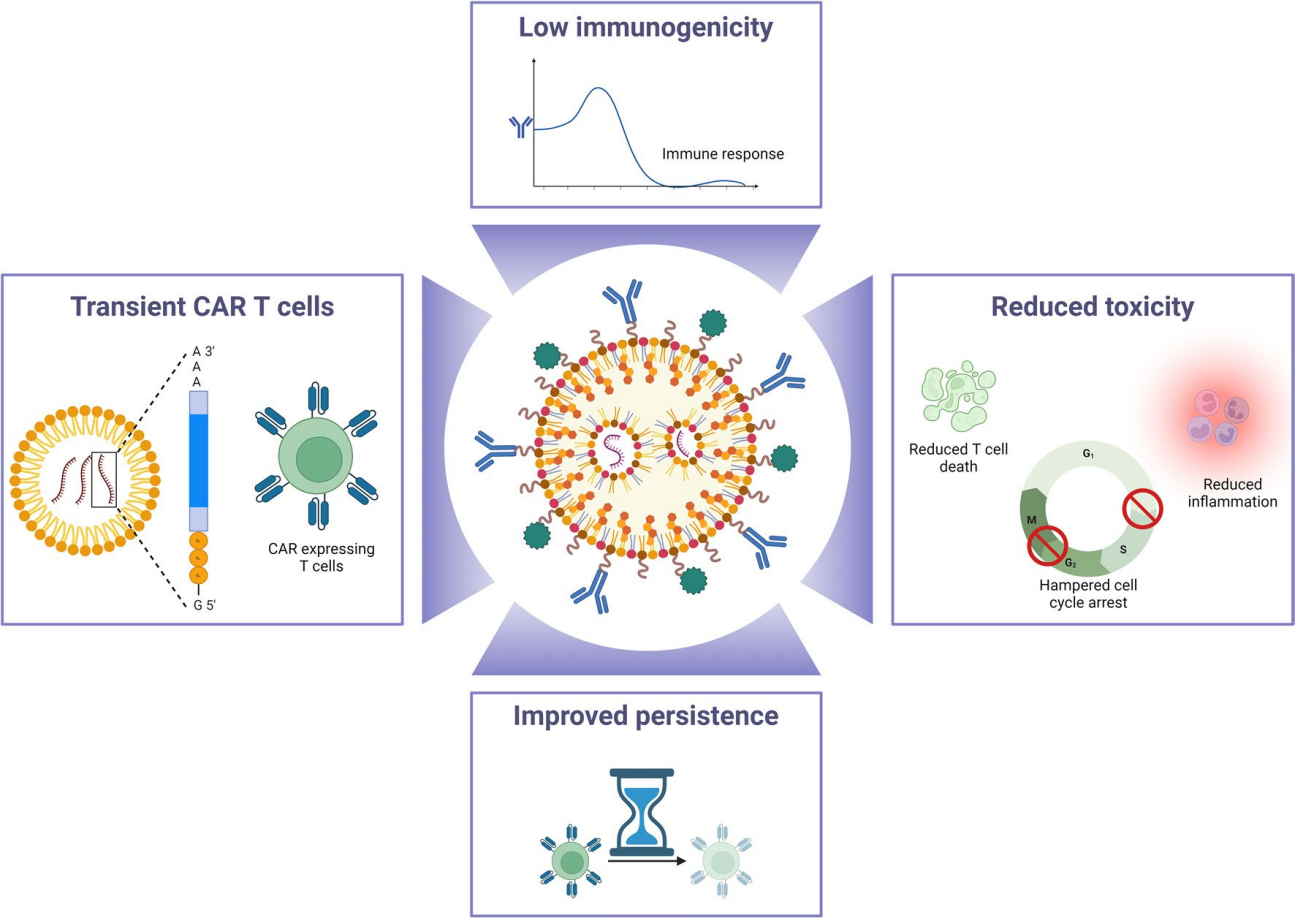

## Introduction

Cancer immunotherapy originated with the hypothesis that the immune system targets tumor-associated neoantigens to prevent carcinogenesis, mirroring graft rejection [1]. Subsequent investigations involving preclinical and clinical assessments of tumor-specific immune responses, along with tumoral adaptive transfers, provided further validation [2]. In the 1990s, the identification of CD4^+^ T lymphocytes mediating the spontaneous regression of melanoma ushered in a new era of adaptive T cell therapy [3]. Currently, the very approach has been revolutionized by adoptive T cell (ATC) therapy via engineered Chimeric Antigen Receptor (CAR) T cells. No doubt the research in clinics concerning CAR natural killer (NK) cell therapy is advancing with a primary focus on augmenting its antitumor efficacy. Research findings underscore the merits of CAR NK cells, including their ability for precise tumor targeting, diverse cell origins, and enhanced effectiveness in combating solid tumors [4–6]. Researchers are actively addressing hurdles such as cytotoxicity, low transfection rates, and challenges related to storage linked with CAR NK cell therapy [7]. On the other hand, CAR macrophages hold promise in cancer immunotherapy as they offer a potential solution to the obstacles encountered by CAR T cell therapy in treating solid tumors [8]. Recent progress has led to the advancement of CAR macrophages into clinical trials [9]. Research has explored the potential of CAR macrophages in reprogramming phagocytic activity against SARS-CoV-2 which has demonstrated encouraging outcomes in viral clearance [10]. The increasing recognition of CAR NK cell therapy's safety and cost-effectiveness has spurred ongoing clinical trials to evaluate its efficacy, while there remains a critical imperative for additional research to address challenges and maximize the potential of CAR macrophages in cancer and infectious disease treatment.

**Fig. 1:**
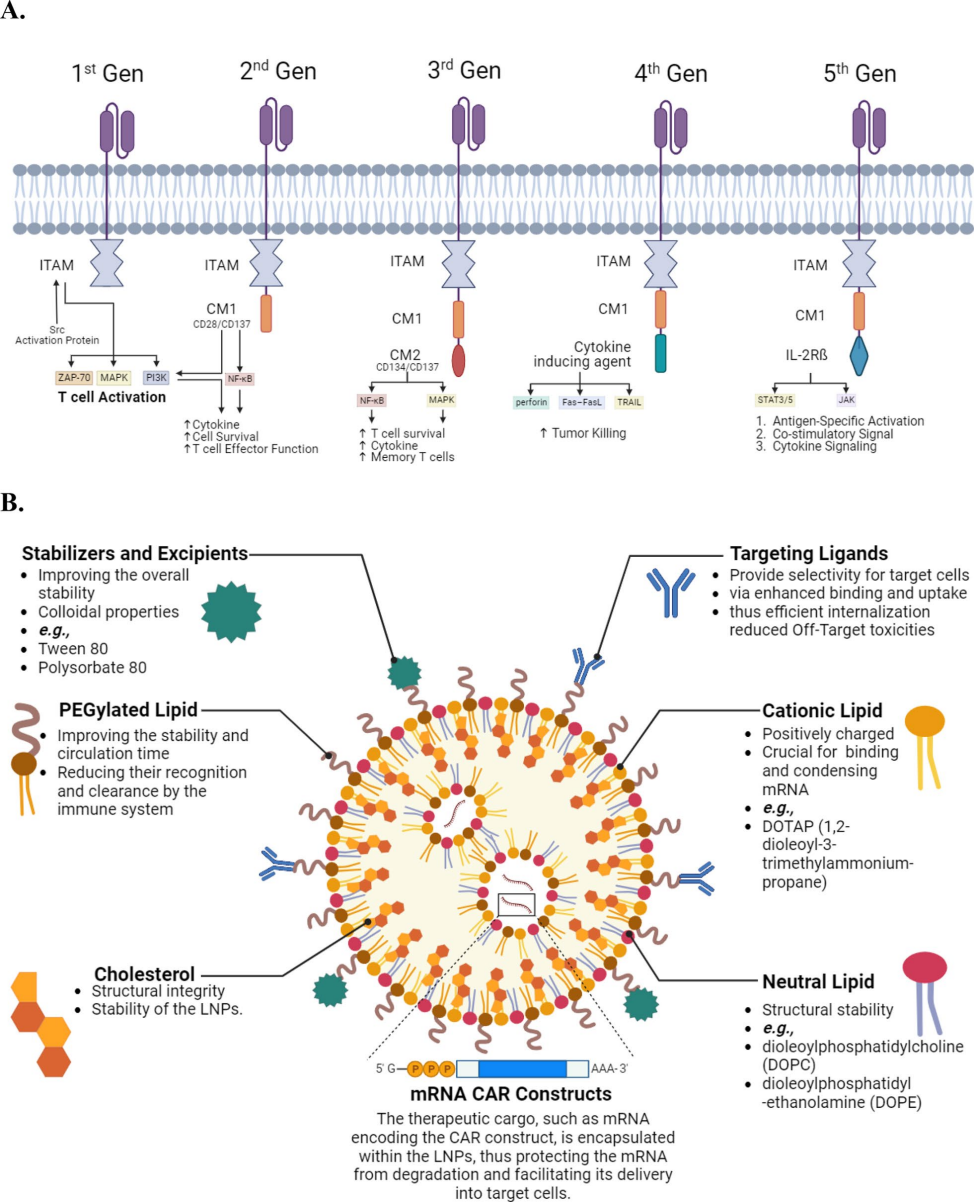
Basic designs of chimeric antigen receptor (CAR) and lipid nanoparticle (LNPs) for CAR delivery.** A** 1st gen CARs rely on immunoreceptor tyrosine-based activation motifs for TCR-associated signaling. 2nd gen CARs enhance proliferation and cytotoxicity by adding CD28 or CD137 co-stimulatory domains. CD28 activates phosphoinositide 3-kinases (PI3K) for improved cytokine production and cell survival; CD137 activates nuclear factor kappa B (NF-κB) pathway. 3rd gen CARs combine CD137 or CD134, activating NF-κB and MAPK for enhanced survival and memory T cell formation. 4th gen CARs secrete desired cytokines, promoting tumor killing via exocytosis or death ligand–death receptor systems. 5th gen CARs, based on 2nd gen, incorporate IL2 receptor β-chain with STAT3 binding, providing antigen-specific activation, T cell receptors (TCR), co-stimulation, and cytokine signals for full T cell activation and proliferation.** B** Positively charged cationic lipids bind and condense mRNA, neutral lipids provide stability, and PEGylation enhances circulation. mRNA, encapsulated in LNPs, protects and delivers the therapeutic cargo. Helper lipids and cholesterol enhance stability, while stabilizers and buffering agents optimize performance. Optionally, targeting ligands improve specificity, promoting binding, uptake, and internalization for enhanced therapeutic precision and reduced off-target effects. Examples include antibodies or peptides which guide engineered T cells to selectively target thereby eliminating cancer cells

To cut to the chase, the victory of CAR T cell therapy in clinical trials is remarkable owing to prolonged response in refractory or relapsed (R/R) hematological malignancies. For example, CAR T cell therapy has demonstrated positive outcomes in clinical success in B-cell acute lymphoblastic leukemia (ALL) (NCT02445248), B-cell lymphoma (NCT02445248) [11, 12], and B-cell maturation antigen (BCMA) (NCT02348216) for multiple myeloma (NCT02658929) [13]. In particular, CAR T cell therapy has gained more than 80% complete response (CR) at initial stages of therapy for B-cell ALL. It has maintained durable responses by establishing immunological memory, resulting in 1-year event-free survival rates of up to 50% [11]. Further, encouraging outcomes are observed in mantle cell lymphoma and R/R follicular lymphoma, with CR rates of 67% and 80%, and progression-free survival rates of 61% and 74%, respectively [14, 15]. Lastly, multiple myeloma registers a CR of 33%, accompanied by a progression-free survival rates of 8.8 months [16]. Given this success, the U.S. Food and Drug Administration (FDA) approves six CAR products, as outlined in Table 1. Earlier, we showcased nanoengineering of better performing CAR T cells [17], mitigating the barriers of the tumor microenvironment (TME) [18] and precision in targeting hematological and solid cancers [19] (Fig. 1A). However, CAR T cells present certain demerits such as; limited T cell trafficking, immunosuppressive environment and antigen escape [20]. Moreover, there are several potential drawbacks and side effects associated with CAR T-cell therapy. For example, cytokine release syndrome and neurotoxicity, [21] which we have reviewed recently and discussed potential insights to mitigate the CRS using CAR T cells for future research [18].

**Table 1:** Overview of FDA-approved CAR T Therapies

CAR product	CAR generation	Approval year	Company	Indication	Target antigen
Tisagenlecleucel	2nd 4-1BB co-stimulatory domain-based	2017	Kymriah®	R/R large BCL	CD19
Axicabtagene ciloleucel	2nd-CD28 based	2017	Yescarta®	Post-first-line therapy; mediastinal large BCL, high-grade BCL, and lymphoma arising from follicles	CD19
Brexucabtagene autoleucel	3rd-Synthetic notch receptor	2020	Tecartus®	Adults with R/R B-cell precursor ALL	CD19
Lisocabtagene maraleucel	2nd-4-1BB co-stimulatory domain-based	2021	Breyanzi®	High-grade BCL, primary mediastinal large BCL, and follicular lymphoma grade 3B	CD19
Idecabtagene vicleucel	3rd-encompassed an immunomodulator, an inhibitor of proteasome, and an anti-CD38 antibody	2021	Abecma®	Adults with MM experiencing R/R status following 4 or more previous lines of treatment	BCMA
Ciltacabtagene autoleucel	3rd-synthetic Notch receptor, encompassed an inhibitor of proteasome, an immunomodulator, and an anti-CD38 antibody	2022	Carvykti®	Adults with MM experiencing R/R status following 4 or more previous lines of treatment	BCMA

MM: Multiple myeloma; BCL: B-cell lymphoma; R/R: relapsed or refractory; BCMA: B-cell maturation antigen

The clinical manufacturing process involves multiple ex vivo stages, including the collection and isolation of T cells from peripheral blood mononuclear cells, followed by activation. This process spans 1–2 weeks [22], thereby impacting and shaping the preclinical outcomes. The pivotal stage is the genetic modification process, accomplished through either viral or nonviral transduction, facilitating the integration of DNA or mRNA. While current market-approved CAR T cells and the predominant focus of clinical investigations employ viral vectors (γ-retroviruses and lentiviruses) for the delivery of the CAR gene [23], it is noteworthy that the intricacies and costliness associated with viral vector production pose considerable challenges. In pursuit of more sustainable and cost-effective approaches, nonviral methods, such as mRNA technologies and transposons, have entered the realm of initial proof-of-concept clinical trials [24]. However, it is crucial to enhance the longevity and safety of these alternative methods through focused research efforts.

In addressing challenges inherent to CAR T cell therapy, particularly toxicities, engineering solutions have gained prominence [25, 26]. Nonviral transduction methods, such as lipid nanoparticles (LNPs), are explored to mitigate toxicities and enhance CAR T cell safety [27] as depicted in Fig. 1B. In this context, Kitte et al. provided experimental evidence by demonstrating an efficient in vitro CAR-mRNA delivery in comparison to electroporation. In contrast to electroporation, LNP-delivered CAR-mRNA showed prolonged in vitro efficacy, thus extended persistence, less toxicity, slower CAR T cell proliferation and less exhaustion [28]. In this review, we offer a thorough analysis of the advantages and mechanistic of LNP-mediated delivery of CAR constructs to improve persistence, efficacy, and mitigating the toxicities. Moreover, we uncover insights into the interactions between LNPs and CAR T cells, current challenges, and their possible solutions.

## Shifting the trend toward lipid nanoparticles

**Fig. 2:**
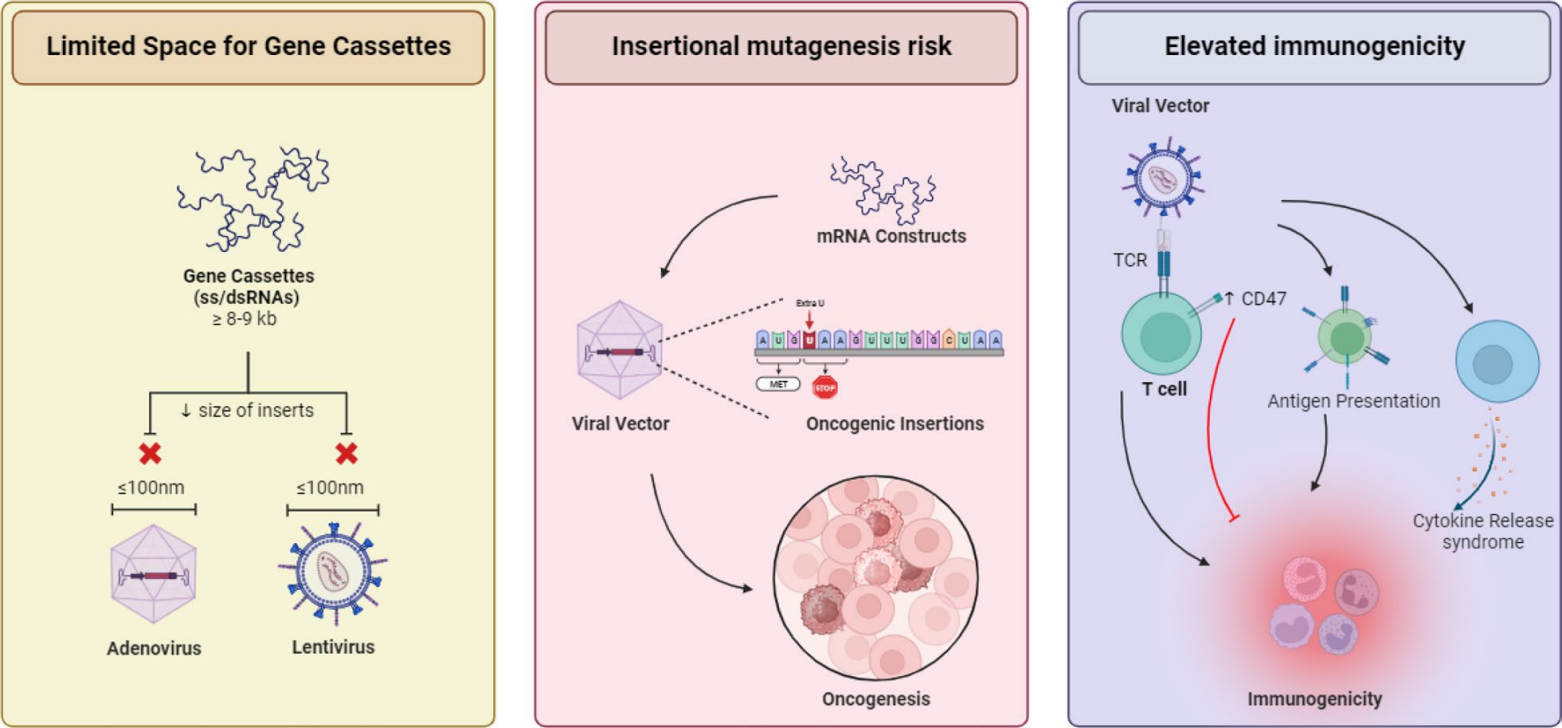
The interplay between capsid size, onco-mutations, and immunogenicity. The constraints stem from the 100 nm diameter of capsids in adenoviruses and lentiviruses, posing challenges for gene cassettes over 8–9 kb. Insertional mutagenesis introduces the risk of oncogenic insertions during construct integration. Viral vectors carry inherent immunogenicity, infecting various antigen-presenting cells (APCs) like DCs, macrophages, or B cells. This prompts APCs to express viral antigens, initiating events culminating in T cell activation and adaptive immune responses. Moreover, viral gene expression induces cytokine production, attracting immune cells and fostering an immune-activating microenvironment

Predominantly, approved CAR T cell products or those under investigation are using viral platforms as a standardized system for delivering the CAR constructs. The main reason for the widespread use of viral vectors is efficient gene transfer and a proven track record of safety in ATC therapy [29]. However, viral vectors face limitations which raises concerns and opens more options to seek. For example, a primary limitation is imposed by the dimensions of viral capsids. The capsids of 100 nm diameter struggle to accommodate gene cassettes exceeding 8–9 kb [30]. This constraint poses challenges when attempting to deliver two different transgenes using separate vectors. Secondly, insertional mutagenesis poses a concern, with the risk of oncogenic insertions during the CAR T cell engineering [31]. Thirdly, utilization of viral vectors comes with an inherent risk of elevated immunogenicity [32]. However, this elevation can be oppressed by overexpression of CD47 thus resulting in the loss of immunogenicity (Fig. 2) [33]. Furthermore, various constraints, including the size of inserts in the virus affecting integration into T cells, extended procedures lasting up to 3 weeks, elevated manufacturing costs, specific responses to virus-derived DNA, restrictions on insert size dictated by capsids, and limited homogeneity among final CAR T cell products [34–36], pose significant barriers to the widespread applications of viral transduction/vectors.

The limitations associated with viral transduction have prompted a shift in research trends toward nonviral methods for gene delivery, seeking alternatives that overcome the challenges posed by viral vectors. One prominent avenue of exploration involves nonviral transduction methods thus offering potential solutions to the constraints associated with viral vectors.

Nonviral methods, such as electroporation and lipofection, provide a safer and more flexible platform for gene transfer without the size limitations imposed by viral capsids [37]. Electroporation, for example, employs electrical pulses to generate temporary pores in cell membranes, facilitating the incorporation of genetic material into target cells. In 2014, Krug et al. and Wiesinger et al. in 2019, independently employed electroporation for good manufacturing practice-compliant production of mRNA-targeted CAR T cells against melanomas [38, 39]. In a recent study by Zhang and collaborators, the utilization of electroporation demonstrated significant success in ensuring both safety and efficacy [35]. This method exhibited a substantial rise in the percentage of memory T lymphocytes within infusion products. Additionally, the study revealed that interference with PD1 positively influenced anti-tumor immune functions, providing further confirmation of the benefits associated with nonviral methods, particularly the integration of PD1 into CAR T cells. Electroporation is versatile to host cells as it swiftly delivers molecules into diverse immune cell types, such as, T cells, dendritic cells (DCs) and CAR T cells, without the need for specific protein targeting or cell tropism limitations [23]. Given these advantages, numerous preclinical ATC therapies incorporate electroporation technology [40–42]. Prominent instances include allogeneic T cell therapies showcasing anti-tumor efficacy in vivo or ex vivo [43]. Moreover, T cell antigen coupler-adoptive immunotherapy has been investigated for cancer treatment overexpressing human epidermal growth factor receptor 2 [42]. However, electroporation is still limited in a sense of dependency on cell type, electrical field strength, and pulse duration thus raising concerns regarding cell viability [44, 45]. For instance, T cells subjected to electroporation with a 25 V waveform displayed a reduced proliferation rate for the initial 2 days post-electroporation compared to control cells [46]. While some studies have demonstrated the potential of electroporation, more clinical data is needed to confirm its safety and efficacy in large-scale applications. For example, Bozza et al. reported the initial clinical trial of virus-free CAR T cells utilizing electroporation [47]. They utilized nonintegrating, compact DNA vectors, devoid of viral components which replicate extrachromosomally, ensuring persistent transgene expression without compromising cell behavior. This strategy enhanced anti-tumor activity in vivo and in vitro when compared to integrating vectors.

Therefore, there is a discernible trend within the research community toward exploring LNPs as an advanced nonviral delivery system. As mentioned earlier, it has been investigated that LNPs beat electroporation owing to their efficient encapsulation and delivery of mRNAs, containing CAR constructs, to target cells [28]. This approach not only circumvents the size constraints of viral vectors but also addresses concerns related to insertional mutagenesis and immunogenicity.

Conclusively, integration of LNPs with CAR technology enhances cancer therapy, particularly CAR T cell development which offer alternatives to viral vectors and addressing challenges like tumorigenicity, complexity, and costs. LNPs enable mRNA delivery, yielding CAR T cells with lower toxicity, comparable efficacy, and reduced expenses as discussed by Kitte et al. [28] and Shin et al. [28, 48]. LNPs extend to CAR NK and CAR macrophage therapies which have shown several benefits e.g., reduced exhaustion, and enhanced anti-tumor responses [28]. LNPs facilitate flexible generation and screening of different CAR T cells thereby enhancing adaptability and efficiency, ensuring safety and cost-effectiveness, and broadening application across cancers and autoimmune diseases.

## Lipid nanoparticles: a versatile drug delivery platform

**Fig. 3:**
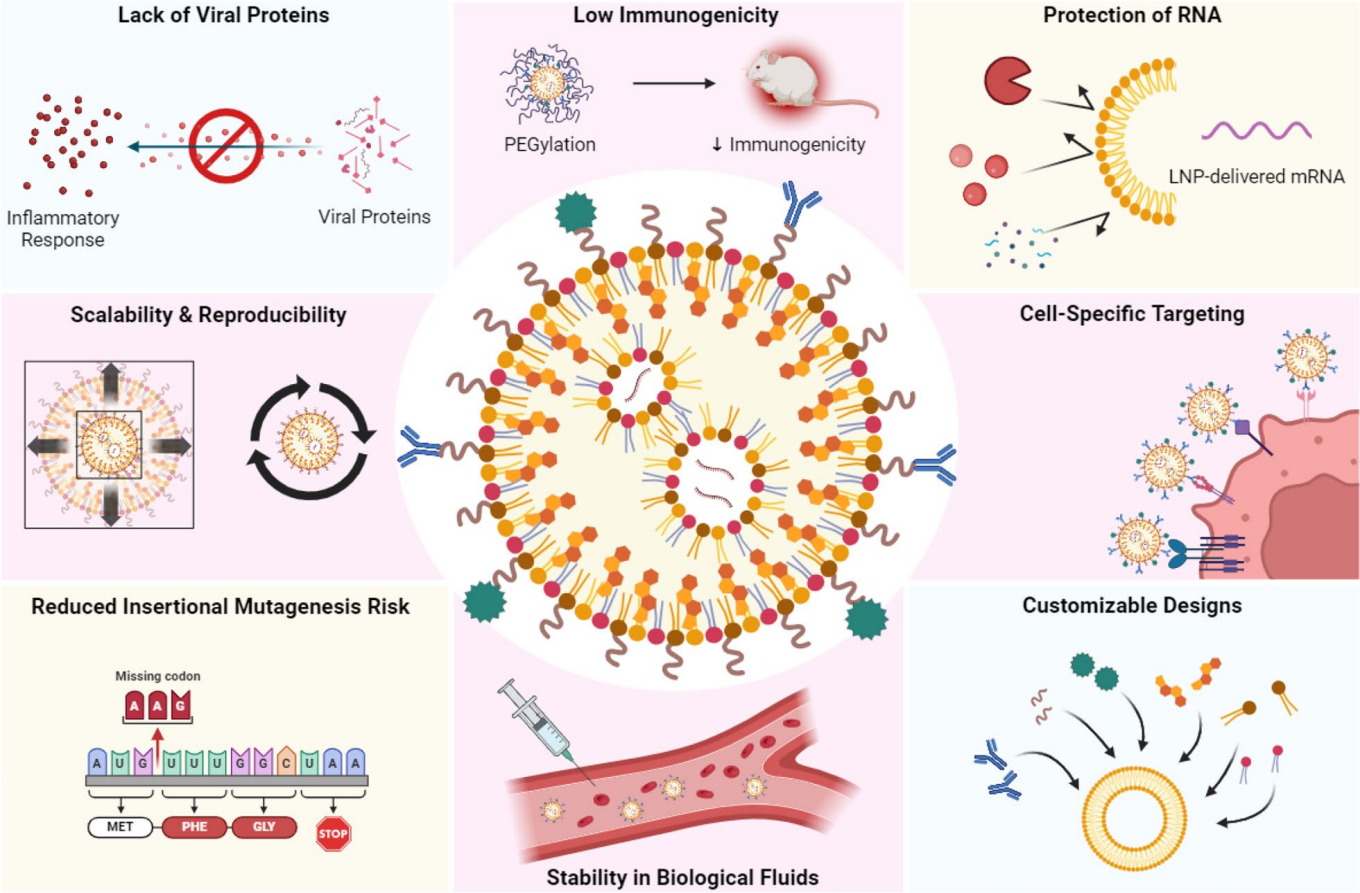
Safety and Efficiency of LNPs in delivering CAR-mRNA constructs. Viral proteins may induce inflammatory responses within host cells, affecting the cellular environment and impacting CAR mRNA delivery success. LNPs, with PEGylation, exhibit lower immunogenicity compared to viral vectors. The mRNA in CAR constructs is protected by a lipid layer, shielding it from endonucleases, cytokines, and insertional mutagenesis. LNPs offer a versatile platform with customizable formulations, allowing tailored lipid composition for specific gene delivery needs. LNPs maintain stability in biological fluids, ensuring genetic payload integrity and improving overall delivery efficiency. Certain LNPs can be engineered for cell-specific targeting, enhancing precision in gene delivery. LNPs scalability and reproducibility support potential translation from research to clinical applications

The use of LNPs in gene delivery is emerging as a promising research direction due to several advantages. For instance, LNPs can encapsulate larger genetic payloads, facilitating the delivery of complex gene cassettes that may be challenging with viral vectors. Li et al. corroborate this claim by introducing a novel technique, multi-laser cylindrical illumination confocal spectroscopy, to analyze mRNA and lipid constituents in LNP formulations at the individual-nanoparticle stage [49]. Additionally, LNPs offer a more controlled and precise delivery mechanism, reducing the risk of insertional mutagenesis associated with viral transduction. They have demonstrated a strong ability to condense and deliver various nucleic acid molecules, spanning in size from small fragments of RNA to entire chromosomes, to cells [50]. LNPs minimize these risks with advantages such as the absence of viral proteins, low immunogenicity, protection of RNA, reduced insertional mutagenesis risk, and efficient mRNA delivery for therapeutic applications [51, 52]. Consequently, their nanoscale size and composition contribute to enhanced biocompatibility and reduced immunogenicity compared to traditional viral vectors. As research progresses, the focus on LNPs in nonviral transfection methods reflects a growing understanding of the need for safer, more efficient, and versatile gene delivery systems regarding CAR T cell therapy. This trend underscores the ongoing efforts to overcome the limitations inherent in viral transduction, aiming to establish novel and improved approaches for engineering safer and effective CAR T cells. However, the effectiveness of gene delivery through LNPs can be impacted by various factors, such as the selection of components and their molar ratios. These parameters significantly affect the stability of nucleic acids within LNPs, and aspects like cellular uptake, endosomal escape and the payload release profile [53] as summarized in Fig. 3.

The potential of LNPs in drug delivery extends beyond gene delivery and encompasses a wide range of applications. Recent mRNA-loaded LNP advancements (as summarized in Table 2) demonstrate precision protein expression, liver-targeted transgene delivery, simplified CAR T cell production, improved mRNA delivery, and promising noncationic thiourea LNPs, requiring further safety and scalability investigations. LNPs have been extensively researched and proposed for various administration routes, making them a versatile and promising drug delivery platform. For prolonged topical drug delivery, innovative LNPs such as solid LNPs [54], nanostructured lipid carriers [55], and micellar nanoparticles [56] have demonstrated significant potential in revolutionizing drug delivery systems.

**Table 2:** An overview regarding potential applications, strengths, and weaknesses of recent studies

Sr	Author	Year	LNP/mRNA modifications	Methodological strategy	Applications	Strengths	Weakness	References
1.	Veiga et al.	2018	Modified mRNA loaded LNPs combined with ASSET	Precision protein expression strategy in Ly6c^+^ inflammatory leukocytes	Targeted expression of interleukin 10 in Ly6c^+^ inflammatory leukocytes in IBD induced mice	Therapeutic alteration of gene expression in vivo	Cell specificity remains a challenge; limited to inflammatory leukocytes	[57]
2.	Di et al.	2022	Firefly luciferase encoding mRNA loaded LNPs	Biodistribution and Luciferase expression levels by bioluminescence imaging and enzyme activity assays	Size dependent biodistribution of LNPs	Transgene expression was most prominent in the liver	Accumulation of large sized LNPs in liver; variable transfection efficiency in different organs; limited biodistribution data beyond liver and spleen	[58]
3.	Álvarez‐Benedicto et al.	2023	SORT LNPs	In situ transfection	Simplified CAR T cell production	Increases overall survival in B-cell lymphoma models; reduces tumor metastasis to liver	No long-term safety profiling; off-target effects of LNPs	[59]
4.	Patel et al.	2022	Substitution of 25% and 50% 7α-hydroxycholesterol for cholesterol into LNPs	Engineering a library of LNPs incorporating hydroxycholesterols	Describes an impact on mRNA delivery to T cells by leveraging endosomal trafficking mechanisms	Enhancing mRNA delivery to T cells, increased late endosome production, reduced presence of recycling endosome	No long-term effects or clinical scalability have been described	[60]
5.	Qiu et al.	2021	Combinatorial synthetic LNPs with distinct chemical structures and properties	Development of bioreducible and biodegradable LNPs using Michael addition reaction	Vaccination, cancer immunotherapy, protein replacement therapy, genome editing	Successful clinical approval of mRNA vaccines and siRNA drug (ONPATTRO) by FDA	Need for specific, efficient, and safe delivery systems; challenges in clinical translation of mRNA-based therapies	[61]
6.	Patel et al.	2024	Bile acid-containing LNPs	Incorporation of bile acids (cholic acid) without cholesterol	Gastrointestinal or immune cell delivery	Generalizability of cholic acid replacement	Optimization needed for large-scale production	[62]
7.	Billingsley et al.	2020	Ionizable LNPs	Screening of a library of 24 ionizable LNPs, selection of top-performing LNP formulation (C14-4)	Potential enhancement of mRNA-based CAR T cell engineering, reduction of cytotoxicity compared to electroporation	LNPs deliver mRNA efficiently to primary human T cells and induce functional protein expression	Further investigations warranted on long-term effects, scalability, and efficiency	[63]
8.	Zhang et al.	2024	One-component ionizable cationic LNPs	Standalone carriers, rational design of cationic lipids rich in secondary amines	Targeted mRNA delivery to spleen and T cells	Efficient mRNA delivery in vitro and in vivo	Investigation of long-term safety and efficacy are warranted	[64]
9.	Wang et al.	2023	Comirnaty®	Biodistribution of PEGylated LNPs and blood clearance	Understanding immune responses to LNPs	Demonstrates time- and dose-dependency of LNP-induced anti-PEG antibodies	Limited to animal model, may not fully reflect human response. Need for human studies	[65]
10.	Wang et al.	2023	Noncationic thiourea LNPs	Strong hydrogen bond interaction between thiourea groups of noncationic thiourea LNPs and phosphate groups of mRNAs	Potential for future mRNA delivery with good inflammatory safety profiles, high gene transfection efficiency, and spleen-targeting delivery for disease treatments	Simplified preparation technology, negligible inflammatory and cytotoxicity side effects, higher gene transfection efficiency in vitro and in vivo, spleen-targeting delivery ability	Further investigation warranted for long-term safety, scalability, and broader applicability beyond spleen targeting	[66]

ASSET: anchored secondary scFv enabling targeting; IBD: inflammatory bowel disease; SORT: Selective ORgan Targeted

## LNPs-mediated nucleic acid delivery

**Fig. 4:**
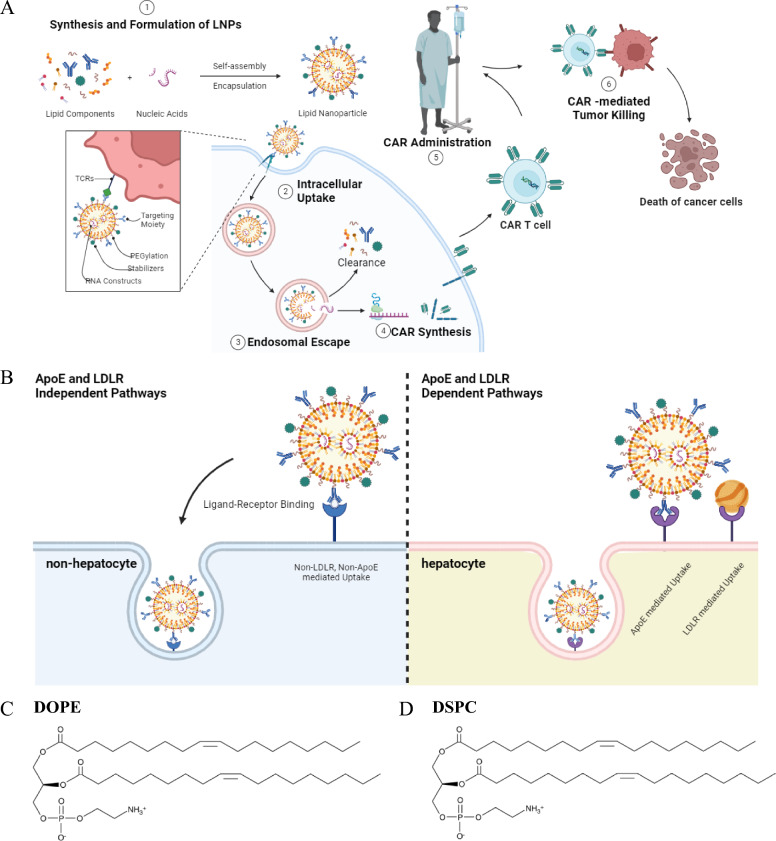
LNPs mediated delivery of nucleic acids. **A** The process begins with formulating LNPs, comprised of lipids, cholesterol, and PEGylated lipids. These self-assemble to encapsulate nucleic acids through electrostatic, hydrogen, and hydrophobic interactions. Stabilizing agents like PEG enhance LNP stability. Intracellular uptake involves endocytosis, facilitated by cell surface receptors. Endosomal escape and cytoplasmic release are crucial for delivering nucleic acids, allowing translation and biological activity. Metabolism and clearance handle unused components. **B** LNPs traditionally target hepatocytes for mRNA delivery. Recent advancements enable LNPs to effectively deliver mRNA to non-hepatocytes, broadening therapeutic targeting beyond liver cells. Progress in ApoE- and LDL receptor-independent pathways enhances the versatility of LNPs in targeting diverse cell types. **C** Structure of 1,2-dioleoyl-*sn*-glycero-3-phosphoethanolamine (DOPE) and **D** 1,2-distearoyl-*sn*-glycero-3-phosphocholine (DSPC)

Using LNPs in CAR T cell therapy presents a promising avenue for overcoming biological barriers associated with nucleic acid delivery. In this context, LNPs are formulated to safeguard mRNAs and facilitate their intracellular delivery [67]. A study used high-throughput in vivo testing to explore the structure–function relations of intravenous (IV) administration of LNPs [68]. Findings indicated that LNPs with helper lipid 1,2-dioleoyl-*sn*-glycero-3-phosphoethanolamine (DOPE) exhibited a predilection for accumulation in the liver, whereas those replacing DOPE with 1,2-distearoyl-*sn*-glycero-3-phosphocholine (DSPC) showed a preference for accumulation in the spleen (Fig. 4C & 4D). Additionally, the study investigated the interaction of LNPs with apolipoprotein E (ApoE) and revealed that DOPE-containing LNPs exhibited enhanced interactions with ApoE than those substituting DOPE with DSPC. Additional confirmation using mRNA and Cy3-small interfering RNA (siRNA) encoding firefly luciferase provided support for improved delivery to particular organs depending on the helper lipid employed. Understanding the impact of helper lipids on the biodistribution and ApoE adsorption of LNPs contributes to the effective design of LNPs for nucleic acid therapeutics. Moreover, LNPs have demonstrated success in delivering therapeutic RNA to hepatocytes, facilitated by ApoE adsorption onto clinical LNP-mRNA drugs [69]. This process entails ApoE-LDL receptor trafficking, which is preserved across species of mice, non-human primates, and humans [70–72]. Recent advancements indicate that LNPs have the capability to transport mRNA to non-hepatocytes through pathways independent of ApoE and LDL receptor, as illustrated in Fig. 4B expanding their potential to target a broader range of cell types [69]. The ability to tune endogenous LNPs trafficking by modifying lipid chemistry opens avenues for enhancing the versatility of LNPs in delivering therapeutic payloads to diverse cell types.

The application of LNPs extends towards extrahepatic delivery, showing promise in T cell therapies, particularly in CAR T cell generation. Optimizing LNPs formulations for mRNA delivery to T cells has demonstrated high transfection efficiency comparable to electroporation. This approach offers an alternative to traditional methods, minimizing the risk of mutagenesis associated with viral vector-based gene transfection [73]. Tanaka et al. emphasizes the importance of optimizing lipid composition for efficient uptake and escape into the cytoplasm, contributing to the development of LNPs as effective tools for transient gene expression in T cells [74].

Improving extrahepatic delivery of mRNA using LNPs presents challenges, such as, achieving sufficient distribution to target tissues and enhancing transfection potency in extrahepatic delivery systems. The observed enhanced protein expression in the spleen and bone marrow with LNPs containing 40 mol % egg sphingomyelin likely results from both prolonged circulation lifetimes and increased transfection potency. However, the achieved circulation lifetimes of 3.7 h are relatively modest compared to DSPC/Chol systems, which can achieve lifetimes exceeding 10 h at high doses [76]. While liposomes composed of bilayer lipid mixtures like DSPC/Chol are well tolerated up to doses of 1 g lipids/kg without adverse effects [76].

Chander et al. demonstrate that LNPs enriched with higher doses of helper lipids like egg sphingomyelin, exhibit enhanced transfection properties both in vitro and in vivo, which extends to extrahepatic tissues without adverse effects. The LNP-system employed in this study, features novel structures with an external lipid bilayer surrounding a solid core in an aqueous interior thereby contributing to improved stability of mRNA cargo and longer circulation lifetimes [75]. Exploring higher lipid doses may thus offer a promising avenue for enhancing LNPs-mediated mRNA delivery to extrahepatic tissues. Just like previously explored in terms of reduced toxicity with improved transfection to T cells [77]; later on, Billingsley et al. explored targeted antibody-conjugated iLNPs with extrahepatic tropism. The LNP formulations used in this study achieved extrahepatic transfection and improved delivery to spleen [78]. Further, Zhang et al. demonstrated the effectiveness of one-component ionizable cationic lipids, rich in secondary amines, for targeted mRNA delivery to the spleen and T cells, overcoming the limitations of extrahepatic delivery. These one-component LNPs exhibit superior mRNA binding and cellular uptake compared to tertiary amine counterparts [64]. This innovative approach offers a simplified and efficient method for mRNA delivery to extrahepatic tissues.

Advancements in understanding the structure–function relationships of LNPs and their interactions with ApoE provide a foundation for tailoring LNPs to specific therapeutic applications, thus, contributing to the advancement of therapeutics based on nucleic acids. The continued development of LNPs holds great potential for overcoming delivery challenges including extrahepatic delivery and slow transfection, contributing to the efficacy and persistence of CAR T cell therapies. Further refining LNP formulations to enhance delivery to specific cell types, including T cells, and improving the efficiency of transient gene expression are warranted.

### Integration of lipid nanoparticles in CAR T cell therapy

Integration of LNPs in CAR T cell therapy holds promise as an alternate to viral vectors and electroporation for engineering CAR T cells. Before applying this strategy, several aspects should be considered such as immunogenicity, type of nucleic acid, reduced toxicity improved safety, scalability, and clinical applicability. Recent research is advancing in using LNPs for all these aspects. For instance, LNPs enable the in vivo production of CAR T cells by transporting therapeutic mRNA to lymphocytes [79], ensuring efficient delivery, lower immunogenicity, and reduced risk of insertional mutagenesis [80]. The scalability of LNPs, with rapid optimization, absence of complex production requirements, and clinical applicability, further position them as a viable option for CAR T cell engineering [81].

### mRNA-centered CAR T cell engineering

Advancements in mRNA-guided CAR T cell engineering have ushered in a new era of innovative approaches, offering unprecedented possibilities for precision and versatility in therapeutic interventions. Among these studies, Hamilton et al. utilized ionizable LNP (iLNP) platform to facilitate concurrent therapeutic gene expression and RNA interference in T lymphocytes. The co-encapsulation of mRNA and siRNA improves expression and knockdown properties, as evidenced by the delivery of CAR-mRNA and PD-1-targeting siRNA, resulting in robust CAR expression and PD-1 knockdown in T cells ex vivo [82].

**Fig. 5:**
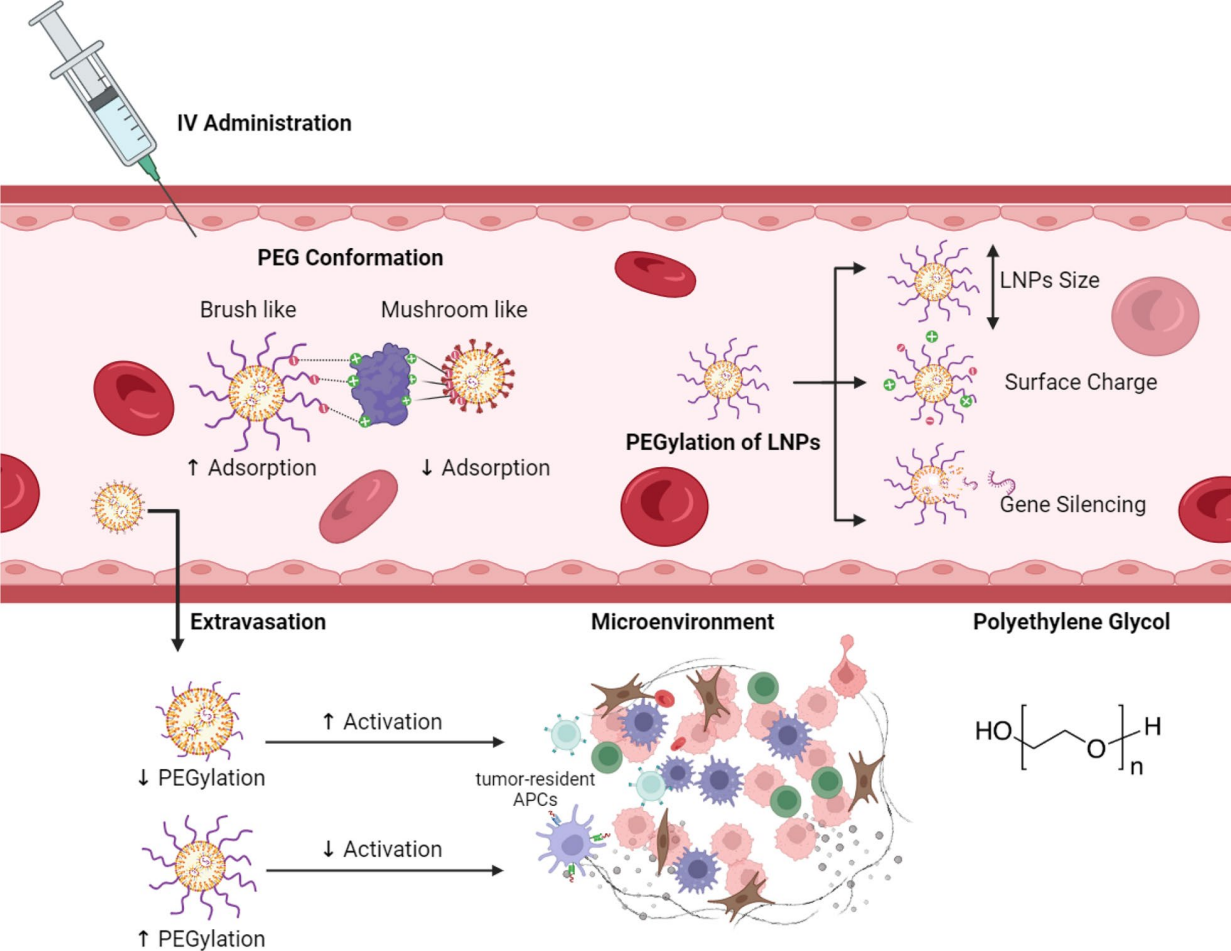
Efficiency of LNPs with PEGylation. Following IV administration, brush-like PEGylation show increased plasma protein adsorption when compared to club-shaped PEGylation or mushroom-like. Also, PEGylation influence LNPs size, its surface charge, and the capability of gene silencing. Next to extravasation, lightly PEGylated LNPs have shown enhanced activation and expansion of tumor resident antigen presenting cells when compared to largely PEGylated has shown reduced effects

Assessing the distribution and payload capacity of mRNA LNPs is essential owing to the molecular assembly mechanisms, pharmacodynamics and kinetics, and delivery efficiency. Further, insights into mRNA packaging characteristics are vital for comprehending the structure–property-function relationships in the development of CAR loaded LNPs. A recent study systematically elucidates a kinetically regulated assembly mechanism that directs payload distribution and capacity in LNPs [49]. Currently, LNPs employed in mRNA vaccines, such as the Pfizer-BioNTech and Moderna COVID-19 mRNA vaccines, include four lipid types: an ionizable lipid, a PEGylated lipid (Fig. 5), cholesterol, and a helper lipid [83]. Owing to the diverse lipid conformations and the intricate nature of self-assembled particles, the structural specifics of mRNA loaded LNPs remain ambiguous. The iLNPs play a crucial role in mRNA interaction and are essential for adjusting the surface charge to control mRNA release into the cytosol through endosomal escape triggered by pH shifts [84] as shown in Fig. 4A. The conformation of PEG is associated with the level of PEG density and found in either sparsely or densely packed configurations [85]. The surface structure of PEG has demonstrated influences on plasma protein adsorption, cellular uptake, in vivo circulation, and other factors [86]. Furthermore, it is important to acknowledge the significance of the surface properties of LNPs, particularly the functional lipid PEG, in improving colloidal stability, prolonging circulation time, and influencing the cellular uptake of mRNA vaccines. In a study by Wang et al. high-field nuclear magnetic resonance (NMR) spectroscopy was used to investigate the composition of lipid at the surface of mRNA loaded LNPs, specifically focusing on identifying the presence of PEG structures and partial ionizable lipids. Utilizing comparative NMR examination across different vaccine formulations and stability samples offers a comprehensive perspective on the external structure of mRNA loaded LNPs, contributing to a more nuanced understanding of product characteristics [87].

Understanding the factors influencing the delivery efficiency of LNPs may provide crucial insights for optimizing mRNA-based CAR T cell engineering strategies. Despite advancements in LNPs designed for intravenous (IV) delivery of siRNA, Hassett et al. underscored the need for tailored LNPs for intramuscular (IM) administration, especially in mRNA delivery, revealing a lead formulation with robust immune responses and improved tolerability. Importantly, heightened innate immune stimulation by LNPs did not correlate with augmented immunogenicity, highlighting the potential to enhance mRNA vaccine tolerability without compromising potency [88]. Successively, NMR, fluorescent-dye binding, and electrophoretic mobility methods unveiled that pKa of iLNPs is 2–3 units elevated than that of the typical LNPs, primarily attributed to variances in proton solvation energy. Consequently, this alteration impacts the negative charge of iLNPs, thereby enhancing off-target systemic mRNA expression in the liver following IM administration [84].

The next consideration in mRNA-based CAR T cell therapy should be the potential concern regarding immunogenicity. As an example, a study establishes a connection between IL-1β, a pivotal cytokine in the innate immune response, and the immunological role of liposomes encapsulating mRNA vaccines [89]. Interestingly, IL-1β secretion did not increase with the treatment of empty liposomes alone in human monocytes; yet, it was amplified in the presence of R848, a toll-like receptor (TLR) 7 and TLR8 agonists. Notably, IL-1β, known for stimulating proinflammatory cytokines, exhibited varied secretion levels depending on the ionizable lipids used. For instance, SM-102-LNPs showed significantly higher IL-1β secretion than MC3-LNPs when comparing modRNA-encapsulated formulations [89]. The innate immune system utilizes a distinct mechanism for LNPs, which is not associated with the NOD-like receptor pyrin domain-containing protein 3 (NLRP3) inflammasome. To explore this further, the innate immunogenicity of the Pfizer-BioNTech COVID-19 vaccine BNT162b2 (Comirnaty) was investigated [90]. The concept that LNPs serve as adjuvants when paired with mRNA vaccines finds support in various investigations. Notably, mRNA containing LNPs are identifiable by TLR, melanoma differentiation-associated protein 5 (MDA5), and NLRP3. Furthermore, mRNA loaded LNPs trigger the release of cytokines such as IL-1β, interferon-gamma (IFN-γ), and interleukin-6 (IL-6) through the innate immunity pathway. Additionally, mRNA loaded LNPs play a role in fostering responses in CD8^+^ T cells, T follicular helper (Tfh) cells, and germinal center B-cells [91]. These observations collectively suggest that LNPs, in conjunction with mRNA vaccines, elicit a multifaceted immune response. Finally, a study focuses on the LNP-triggered immune response and highlights the time- and dose-dependency of LNP-induced anti-PEG antibodies. The administration of LNPs resulted in an unforeseen isotype switch and the development of immune memory, resulting in a swift boost and prolonged duration of anti-PEG IgM and IgG upon re-administration in rats. Significantly, the initial LNPs injection expedited the clearance of subsequent doses in the bloodstream of rats [65]. This understanding is crucial for elucidating potential immune reactions associated with clinically relevant LNPs.

Combinatorial alterations in LNPs offer valuable insights into the incorporation of LNPs in CAR T cell therapy. By excluding ligands, piperazine-containing iLNPs (piLNPs) demonstrated a preference for the delivery of mRNA to immune cells in vivo. High-throughput DNA barcoding assesses 65 LNPs, revealing insights into lipid structure, cellular targeting, and identifying traits enhancing in vivo delivery. Notably, at a clinically relevant dose of 0.3 mg/kg, pi-A10, an LNP, exhibits a predilection for delivering mRNA to the liver and immune cells in the spleen [92]. These findings underscore the potential of in vivo studies to identify LNPs in a variety of cells, supporting the use of bioactive small-molecule motifs in mRNA delivery, and providing valuable insights for LNPs integration in CAR T cell therapy. These findings, shedding light on the adjuvant properties of LNPs in the context of immunogenicity, have implications for the advancement of mRNA-based CAR T cell engineering strategies.

### Transient CAR T cell generation

LNPs have the capability to produce CAR T cells in vivo, providing the opportunity for systemic application to generate CAR T cells directly within patients. This approach can be especially advantageous for specific therapeutic applications. Since mRNA is confined to the cytoplasm, unable to genomic integration, inherently unstable, and diluted during division, the CAR T cells generated using LNPs are transient [93]. This is an important feature, as non-integrative systems can help limit off-target effects and toxic effects in the long-term [94]. The traditional ex vivo handling of T cells for CAR T cell therapy frequently relies on viral vectors, resulting in permanent CAR expression and the possibility of adverse effects. Billingsley et al. explored the use of iLNPs for ex vivo mRNA delivery to T cells as an alternative strategy. Their study synthesized a library of 24 iLNPs, with the top-performing LNP, C14–4, showing enhanced mRNA delivery and lowered cytotoxicity [63]. The platform effectively transported CAR-mRNA to T cells, eliciting CAR expression comparable to electroporation but with markedly reduced cytotoxicity. The engineered CAR T cells exhibited robust cancer-killing activity, highlighting the capability of LNPs to improve mRNA-based CAR T cell engineering with transient expression. This approach aligns with the broader trend of exploring LNPs to improve mRNA therapeutics, as exemplified by Hamilton et al. who developed LNPs for simultaneous therapeutic gene expression and RNA interference, showing potential in achieving transient gene silencing in T cells [82]. Additionally, Ye et al. effectively engineered CAR macrophages and CAR T cells using LNPs for in vitro mRNA transfection, providing a glimpse into the potential of LNPs for cost-effective and safe mRNA-based adoptive cell therapy [95]. These studies collectively underscore the evolving landscape of transient CAR T cell generation, highlighting LNPs as a promising tool in immunoengineering applications for improved cancer immunotherapies.

Further, in the pursuit of transient CAR T cell generation, studies have explored alternative delivery methods to mitigate potential drawbacks associated with sustained expression. A study demonstrated the transient expression in human induced pluripotent stem cells (hiPSCs) via one-step delivery of CRISPR-Cas9 components, resulting in a high indel rate of up to 85% at multiple loci. Notably, allele-specific sgRNA targeting compound heterozygous mutations exhibited a preference for interallelic gene conversion, showcasing the versatility of LNPs in achieving targeted transient modifications [96]. Further, a study by Zhao et al. addresses a significant challenge of transfection of larger biologics such as mRNA, to primary T lymphocytes. By systematically screening and optimizing a library of lipidoids as potential delivery vehicles, the study pinpointed imidazole-containing lipidoids that exhibited potent transfection capabilities in T lymphocytes. Notably, the lead lipidoid was utilized as a vehicle for Cre mRNA in vivo, resulting in a commendable genetic recombination of 8.2% in mice T cells [97]. Therefore, the efficient mRNA delivery to T lymphocytes is a crucial aspect of transient modifications. Further exploration and optimization of such LNPs may pave the way for enhanced transient modifications in CAR T cell therapy.

The context of transient CAR T cell generation via LNPs involves addressing challenges associated with traditional methods, such as in vitro transfection, that lead to prolonged persistence, side effects, and complex isolation processes. To address these roadblocks, Rurik et al. proposed immunotherapy for cardiac fibrosis using CD5-targeted LNPs to deliver mRNA constructs, achieving in vivo production of transient CAR T cells while reducing fibrosis thereby restoring cardiac function [98]. Further, Tombácz et al. focused on the T cell resistance to transfection by developing CD4-targeted LNPs for specific mRNA interventions in CD4^+^ T cells [99]. This targeted approach enabled efficient transfection of T cells, providing a platform for immunotherapy of various conditions. Moreover, Paunovska et al. presented the fast identification of nanoparticle delivery (FIND) system to assess LNPs in delivering the mRNA. They identified an LNP with oxidized cholesterol that efficiently delivered Cre mRNA to liver microenvironmental cells, showcasing the potential for gene editing in clinically relevant doses [100]. Further developing PEG-free RNA therapeutics, Nogueira et al. explored polysarcosine-based LNPs for mRNA delivery, demonstrating controlled particle engineering and improved safety profiles compared to PEGylated LNPs [101]. Collectively, these studies address challenges in transient CAR T cell generation by presenting innovative solutions such as in vivo mRNA delivery using targeted LNPs, efficient transfection of resistant T cells, and controlled particle engineering for enhanced safety and efficacy. These insights pave the way for future developments in the field, promising more effective and safer transient CAR T cell therapies.

### Reduced toxicity and improved safety

LNPs have shown promise in reducing T cell toxicity while maintaining comparable levels of CAR surface expression in contrast to electroporation. This suggests the potential of LNPs to improve methods for engineering mRNA-based CAR T cells. Several challenges persist in in vitro gene editing of T cells and hematopoietic stem/progenitor cells (HSPCs), a promising avenue for treating cancers. For example, electroporation induces significant cytotoxicity in T lymphocytes, initiating cell death, cell cycle interruption, and inflammation [102]. Meanwhile, there are certain limitations of current mRNA delivery systems to primary T lymphocytes [103]. However, efficiently manipulating T lymphocytes through gene delivery has played a crucial role in the execution of diverse immunotherapies [104], immune checkpoint blockade [105], and in situ T cell reprogramming [106]. Furthermore, there is a need for future research to investigate the inefficiency of immunosuppressive therapies. To address these hurdles, Vavassori et al. demonstrated that nuclease RNA delivery through LNPs significantly reduces cell death, ameliorates cell growth, and enhances tolerance in T cells. Further, LNPs, as shown by Li et al., suggest a promise for the delivery of mRNA in primary T cells, with enhanced transfection efficiency and selective spleen tropism. Finally, Thatte et al. demonstrated the creation of an LNP platform for the efficient delivery of Foxp3 mRNA to CD4^+^ T cells. This successful engineering results in immunosuppressive T cells with transient phenotypic expression.

In light of the importance of specific antibody modifications, we assert that antibody modification constitutes a fundamental approach in the development of immunocyte-targeting LNPs. For example, the conjugation of CD4 antibodies to LNPs facilitates precise targeting and mRNA interventions in CD4^+^ T cells [99]. Furthermore, IV administration of CD4-targeted LNPs loaded with Cre recombinase-encoding mRNA results in specific genetic recombination in CD4^+^ T cells within the spleen and lymph nodes [91]. Moreover, the conjugation of antibodies targeting pan-T cell markers expands the scope of T cell targeting with LNPs [107]. By tailoring the composition and characteristics of mRNA loaded LNPs, such as through the use of adjuvants and regulation of injection routes, we can modulate their immunogenicity [78]. Overall, we posit that integrating antibody modifications into LNPs represents a promising strategy for precise mRNA payload delivery to modulate T cell function, with the potential for further optimization to enhance targeting and immunogenicity. Incorporating specific antibody modifications into LNPs not only enhances targeted mRNA delivery to modulate T cell function but also contributes to reduced toxicity and improved safety.

Nevertheless, the in situ transfection of T cells using anti-CD3-targeted LNPs (aCD3-LNPs) exhibited effective delivery of mCherry mRNA to Jurkat T cells through targeted LNPs. T cell activation and exhaustion were related to the presence of the aCD3 antibody via superficial coating on LNPs. Additionally, when employed in mice with tumors, aCD3-LNPs facilitated the localization of transfected T cells within tumors and tumor-draining lymph nodes during immunotherapy [108]. Additional approaches might involve the optimization of LNPs structures, examination of supplementary lipid components, and mitigation of potential adverse effects. A critical aspect in translating these discoveries into viable and safe CAR T cell therapies lies in investigating the long-term effects and clinical applicability of LNP-based approaches across diverse therapeutic contexts.

### Improving CAR T cell persistence and efficacy

Recent studies showcase the capability of LNPs to augment mRNA delivery, enhance T cell transfection, and optimize the immunogenicity of mRNA-based therapies. These findings open up new possibilities for the development of targeted and efficacious CAR T cell therapies.

Enhancing the persistence and effectiveness of CAR T cells poses several challenges, and researchers are investigating innovative solutions utilizing LNPs in various studies. One obstacle is the intricate in vitro programming of T cells, a process that is both labor-intensive and costly. To address this challenge, anti-CD3-targeted LNPs have been developed for the in situ transfection of T cells, effectively delivering reporter gene mRNA to achieve T cell activation and depletion [108]. This targeted approach not only demonstrated successful transfection in vitro but also showed promising results in vivo, with LNPs accumulating in spleen and transfected T cells localizing within cancer cells following immunotherapy. Further, the COVID-19 epidemic prompted the quick development of mRNA vaccines, and the success of LNP-formulated vaccines highlights their therapeutic potential. Extending this concept to CAR T cell therapy, LNPs offer a versatile platform. Charge-altering releasable transporters (CARTs), a class of LNPs, has been explored for their effective delivery of mRNA [109]. These CARTs, when functionalized with a small-molecule drug like fingolimod, demonstrate superior transfection of lymphocytes, showcasing the potential for targeted and enhanced mRNA delivery to T cells. Additionally, the development of mRNA vaccines using LNPs, such as CARTs, has demonstrated the utility of LNPs in inducing a robust immune response [110]. This approach offers a promising alternative to traditional methods and suggests the flexibility of LNPs in enhancing the immunogenicity of mRNA vaccines. Another study involving Pi-lipids further expands on the potential of LNPs, showing their preference for mRNA delivery to various immune cells in vivo without requiring ligands targeting [92]. High-throughput in vivo investigations with Pi-lipids identified specific lipid traits that enhance mRNA delivery, emphasizing the role of LNPs in optimizing mRNA-based therapies. Studies have highlighted the effectiveness of cationic liposome-based nanoparticles in protecting mRNA and enhancing delivery [111–113]. LNPs with encapsulated self-amplifying RNAs (saRNAs) have demonstrated increased immunogenicity compared to unformulated RNA, showcasing their potential for therapeutic applications. Notably, LNPs can be formulated with saRNAs either interiorly or on the surface, presenting a novel approach [113]. The exterior complexation of LNPs with saRNAs provides several advantages, including the ability to perform comprehensive quality control on LNP batches before incorporating saRNAs. This approach enhances flexibility in engineering LNPs with different RNA constructs, facilitating rapid formulation for targeting epidemic outbreaks.

Thus, an optimization study demonstrated electrostatic adsorption of large biotherapeutics, such as asRNA to the surface of LNPs which requires protection from degradation and efficient cellular uptake [114]. Although LNPs have been extensively employed for diverse RNA formulations, a dominant paradigm typically revolves around encapsulating RNA within the particle. However, a comparative study assessing LNP formulations with cationic and ionizable lipids challenges this paradigm. Formulating saRNA on the surface of cationic LNPs emerges as an effective substitute, providing protection against RNAse degradation even when adsorbed to the surface [114]. Cationic LNPs demonstrate equivalent in vivo and ex vivo saRNA delivery, inducing comparable antibody responses. Enhancing CAR T cell efficacy confronts challenges, particularly in ex vivo gene editing. Recent studies propose a solution: whereas, LNPs minimize cell death, enhance cell growth, and improve overall tolerance, yielding more edited cells than electroporation [115]. LNPs also support greater clonogenic activity and comparable or superior reconstitution by long-term repopulating HSPCs, emphasizing their potential in improving ex vivo gene editing for CAR T cell therapy.

Overcoming challenges in CAR T cell therapy is crucial for achieving sustained and effective responses. One obstacle involves the effective priming and amplification of T cell responses within lymphoid organs. Addressing the need for persistent and effective responses in CAR T cell therapy poses a significant challenge. Efficient priming and amplification of T cell responses within lymphoid organs are critical for success. An innovative solution is presented in the form of RNA-lipoplexes, utilizing lipid carriers. This approach precisely targets DCs in vivo, ensuring efficient uptake and expression of encoded antigens. The result is the initiation of IFN-α release and the induction of robust effector and memory T cell responses, demonstrating its potential for cancer immunotherapy [116]. Intracellular mRNA delivery for therapy faces complexities, and optimizing LNP formulations is crucial. Design of experiment methodologies has been employed to develop a generalized strategy for optimizing LNPs for mRNA delivery in liver. An optimized formulation demonstrated a sevenfold increase in potency, emphasizing the importance of lipid ratios and structures. Interestingly, the enhanced formulation did not result in improved siRNA delivery, underscoring distinctions in design spaces between siRNA and mRNA [117].

Current investigations emphasize the potential of LNPs in overcoming obstacles related to CAR T cell persistence and efficacy. Optimized LNP formulations enhance the potency of mRNA delivery, providing insights into overcoming intracellular delivery challenges. The integration of LNPs in CAR T cell therapy shows promise for addressing these obstacles and advancing the field toward improved persistence and efficacy.

## Barriers to overcome in LNPs-mediated delivery

Initiating effective LNPs-mediated mRNA delivery encounters various obstacles, necessitating considerations for administration routes, physiological barriers, and design specificities to achieve precision targeting. Beyond these fundamental aspects, several additional tumors associated barriers, discussed in sections hereafter, impede the seamless deployment of LNPs. Recognizing the importance of dealing with these barriers is crucial for making progress in using LNPs to deliver CAR-mRNA constructs.

### Gene transfection related barriers

Within LNPs-mediated mRNA delivery, gene transfection into immunocytes presents a distinct set of barriers. Particularly, immunocytes pose unique challenges such as; systemic inflammatory responses, inefficient cell-specific delivery, instability, and degradation of mRNA within LNPs, and supply chain issues that demand specialized strategies for efficient and targeted gene delivery.

A careful analysis of a recent study by Chen et al. [118], where they employed LNPs to endogenously target lymph nodes, revealed several factors such as; chemical structure of LNPs, optimization of the LNP formulations, undesired expression in non-lymphoid organs and immunogenicity of LNPs, which limit T cll transfection. For example, shorter tail lengths (O10B and O12B) in LNPs exhibit higher mRNA expressions, replacing the ester bond with an amide bond significantly decreased transfection in lymph nodes, and modifying the methyl groups of the amine head to other groups such as hydroxyl, ethyl, or N-(1,2-ethanediyl) acetamide groups reduced mRNA expression. Furthermore, the top-performing lipid (113-O12B) faced challenges in further optimizing the formulation for enhanced efficacy. The formulation needed to be carefully adjusted for components like active lipid, helper lipid, and PEG to achieve desired mRNA transfection levels. Finally, the undesired expression of mRNA in nonlymphoid organs, such as the liver, poses a risk and reduces the specificity of delivery [118]. Therefore, ensuring the targeted delivery of mRNA to lymph nodes rather than nonlymphoid organs is crucial for reducing side effects and improving efficacy.

Interestingly, it has been noted that even when uptake occurs, it does not guarantee successful gene delivery [119]. The reason for this unsuccessful gene delivery was later suggested as that endosomal acidification is slower and less robust in human T cells compared to HeLa cell lines [120]. This suggests that future investigations should not rely on pH-triggered release for successful transfection. Small size, dynamic membrane properties, and intracellular environment pose challenges in transfecting T cells [121]. To address these challanges, Ramishetti et al., formulated targeted LNPs by incorporating various designs whereby these LNPs showed specificity by targeting only primary T cells instead of nonimmune cells. Moreover, upon IV administration, these LNPs showed effective binding and efficient uptake in spleen, blood, lymph nodes, and bone marrow [122]. These findings suggest that traditional LNPs may lack sufficient stability or efficiency when delivering genetic material into T cells. This study further notes that gene silencing via targeted LNPs occurs in a subset of circulating and resting CD4^+^ T lymphocytes [122]. This observation suggests that LNPs may have varying efficacy depending on the activation state or functional status of the target T cells.

Tombácz et al. used CD4-targeted LNPs to deliver Cre mRNA specifically to CD4^+^ T cells in vivo, resulting in a significant increase in ZsGreen1-expressing cells compared to control LNPs. They tested a range of mRNA doses to optimize transfection efficiency, observing higher responses with targeted LNPs. However, the highest dose was found to be toxic. Finally, selective CD4 targeting did not increase nanoparticle uptake in macrophages and DCs, likely due to their extensive natural phagocytic uptake of nanoparticles. However, there was a significant increase in targeted mRNA-LNPs uptake compared to untargeted control mRNA-LNPs in CD4^+^ T cells [99]. Another approach utilized by McKinlay et al. demonstrated that the CARTs show 9-folds efficient translation in lymphocytes, while hybrid-lipid CARTs with optimized ratios of lead lipids demonstrated comparable delivery efficacy to noncovalent mixtures thereby enhancing lymphocyte transfection in primary T cells and in vivo [123]. Further studies have tried to overcome barriers in transfecting B lymphocytes [124] and inflammatory leukocytes [57] and achieved a significant success.

In summary, we propose developing specialized LNPs to overcome barriers in immunocyte transfection, optimizing formulations to enhance mRNA expression and minimize immunogenicity, and exploring innovative targeted delivery methods to specific immune cell populations while reducing off-target effects. Meanwhile, it is crucial to explore alternative gene delivery mechanisms beyond pH-triggered release, given the slower endosomal acidification in human T cells and challenges posed by their small size and dynamic membrane properties. Lastly, we suggest refining the targeting strategies like CD4-targeted LNPs or CARTs to improve specificity and uptake efficiency in target immune cells while minimizing uptake by non-target cells like macrophages and DCs. Further efforts are warranted to overcome barriers in transfecting other immune cell types beyond T cells, such as B lymphocytes and inflammatory leukocytes, to broaden the applicability of LNPs-mediated mRNA delivery in immune-related disorders.

### Administration routes and organ distribution barriers

Generally, nanoparticles encounter difficulties reaching their target as they must navigate through various barriers to achieve effectiveness. While injection is often considered for targeted nanoparticles [125], oral administration has historically been considered impractical [126] despite being the most commonly used method. Drugs administered orally encounter obstacles such as tight junctions, mucus, digestive juices, immune components, and microbial substances in the intestinal barrier [127]. Following oral administration, particles may leak through lymphatic vessels, entering the systemic circulation. Non-intravascular methods, like IM injections, encounter barriers such as extracellular and vascular endothelial obstacles before reaching systemic circulation [128].

Organ distribution highly depends on the route of administration and understanding how LNPs distribute throughout the body is crucial for effective administration. For example, when mRNA-LNPs were administered through various routes in vivo, evidence indicated widespread mRNA activity in all injected regions and most of routes showed systemic spread in mice, however, intradermal and subcutaneous injections exhibited localized activity. Furthermore, delivered mRNA-LNPs demonstrated consistent protein levels 5–10 days post-administration [129]. From this evidence, it is obvious that mRNA-LNPs can reach other organs and tissues via systemic circulation. However, the proliferation of smaller LNPs was more than that of LNPs of larger size. Moreover, further RNA activity may be affected by the biodistribution and pharmacokinetics of LNPs [58]. IV-administered LNPs accumulate in liver and there has been an increased uptake of LNPs by hepatocytes [130] which greatly limits the delivery efficiency of LNPs to other organs. As mentioned earlier, Chen et al., 2020 introduced an additional component in the structure of LNPs for selective organ targeting (SORT) to delivery mRNA for therapeutic editing of non-hepatic cells via IV administration [70]; however, the detailed insights in its mechanism remained to be elusive. The same group, later on, demonstrated mechanistic insights on how SORT-LNPs beat the barrier of liver accumulation. Notably, PEGylated LNPs acquire enhanced colloidal stability [131] and thus its desorption from LNP surface exposes the SORT and enables it to bind with transport proteins in serum. Consequently, this interaction of organ specific SORTs with LNPs enables specific targeting to various organs by promoting cellular uptake. Moreover, mRNA delivery via LNPs with anionic or cationic components to non-hepatic cells is mediated by ApoE independent pathway [132]. Additional to liver, LNPs have been reported to accumulate in lymph nodes following IV administration [133] which may enhance immune response [134]. For example, when mRNA and TLR4 agonist were intravenously co-delivered using LNPs, Th1 immune response was stimulated and tumor suppression with immune memory was observed [135]. Moreover, LNPs mediated mRNA in vivo delivery to spleen stimulated strong CD8^+^ T lymphocyte [118] and T follicular cell responses [136]. Despite the stimulation of antitumor immune responses as evident in these studies, challenges persist. For instance, while providing immune memory and robust T cell responses, the intricate interplay with the immune system may pose hurdles in achieving optimal mRNA delivery via LNPs. Therefore, precise control over immune responses and addressing other associated challenges are crucial for optimized LNPs-mediated mRNA delivery. While SORT-LNPs, formulated through scalable synthetic chemistry and engineering protocols, provide a versatile and precise approach for targeting organs beyond the liver, achieving cell-type specific delivery remains a challenge.

Recently, an LNP-based mRNA delivery platform was engineered for hepatic reticuloendothelial targeting, which demonstrated enhanced mRNA expression with a single lipid change in formulation of Onpattro to induce anionic charge on the LNP surface [137]. The recent development of a peptide in hepatocellular carcinoma (HCC), introduces a novel active targeting strategy for mRNA-based HCC therapy [138]. This contrasts with the modulation of Onpattro formulation [137], and chemical conjugation of ligand to add a targeting moiety [98], emphasizing the need for specific targeting in liver diseases. Additionally, modulating lipid composition can be another strategy to target specific organs, as shown by the addition of anionic or cationic components in LNPs utilizing ApoE independent pathway [132] and via addition of SORT molecule [70].

Nonetheless, potential challenges may arise in achieving precision targeting in diverse liver-related conditions, and a promising solution lies in exploring combinatorial approaches that integrate both cancer-specific peptides and LNP-based strategies for improved efficacy across a spectrum of liver disorders. Nonetheless, advancing LNP engineering for liver targeting requires new systems to access non-liver tissues, emphasizing ongoing research on design rules for reduced liver uptake and effective non-hepatic targeting, notably achieved in lungs and lymphoid tissues [139]. Several strategies have been investigated for lungs, for example, LNPs with modification in GALA peptides [140], modified anti-PECAM1 [141], anti-PV1 [142], and ionizable lipids with amide linkages [143] and anionic lipids [132].

### Extracellular barriers

Despite significant progress in LNP-mediated mRNA delivery, challenges persist in fully overcoming post-administration barriers before reaching target cells. While systemic spread after IV injections has been discussed earlier, issues like liver and spleen accumulations remain terra incognita. Ongoing research should address extracellular barriers, including premature degradation, limited cellular uptake, endosomal escape compatibility, innate immune responses, extravasation, and protein corona interactions [144]. Additionally, understanding the physiological complexities of the gastrointestinal tract is crucial for effective oral administration, suggesting avenues for research to enhance overall delivery efficiency. Therefore, a successful delivery vehicle must navigate the entire process, resisting pre-mature degradation, evading immune surveillance, avoiding non-specific bindings with serum proteins, preventing renal filtration, facilitating extravasation to targeted tissues, and aiding in membrane crossing.

The issue of premature degradation poses a challenge to mRNA delivery through LNPs, compromising mRNA stability post-administration. To counteract this, it is crucial to encapsulate and safeguard the mRNA within LNPs, preventing degradation and facilitating efficient delivery to the cellular cytosol [145]. Incorporating PEG lipids in LNPs contribute to shelf stability by preventing aggregation and leakage of the mRNA payload during storage [145]. Strategies have been implemented to enhance the in vivo stability of mRNA by optimizing its structure, with successful outcomes achieved through encapsulation in LNPs [146]. In overcoming challenges related to premature degradation, lyophilization emerges as a method to enhance the stability of mRNA-LNP formulations, enabling potential storage at elevated temperatures [145]. However, this approach necessitates reconstitution before administration and involves substantial costs. Despite these considerations, studies indicate that LNPs stand out as advanced carriers for mRNA delivery, offering protection against premature degradation [146]. Ensuring the stability of both LNPs and their components is pivotal for efficient mRNA delivery, particularly given the long-term storage demands associated with the global distribution of vaccines [145]. To address the challenge of premature degradation in systemic circulation, it is essential to encapsulate mRNA within LNPs which are capable of safeguarding it from degradation and bolstering stability for effective delivery to target cells. Ongoing efforts, including the use of PEG lipids and exploring lyophilization, aim to enhance the stability and efficacy of mRNA-LNP vaccines, providing potential solutions to improve the overall performance of these vaccines.

Size Does Matter—LNPs (> 100 nm), following IV administration, tend to accumulate in liver while LNPs (< 100 nm) escape the blood vessels via pores in endothelium [147]. Interestingly, LNPs (< 50 nm) show deep penetration and persistence in TME [148], while, LNPs (20–200 nm) are more inclined to be taken up by DCs [149] but LNPs (500 nm–5000 nm) are more vulnerable to phagocytosis by macrophages irrespective of route of administration [150], also LNPs (1-10 μm) are prone to clearance from blood thus, generally, are not recommended. Given that larger LNPs are susceptible to phagocytosis by the first line immune cells, their application becomes more significant in immunotherapy. For instance, the systemic delivery of mRNA-loaded LNPs (≥ 200 nm) in a microfluidics study demonstrated increased activation gene expression in vivo, specifically targeting lymphocytes and DCs in the spleen [151]. Moreover, varying LNP size in a constant lipid composition impacted immunogenicity differently in murine and non-human primate models. While murine responses exhibited size-dependent trends, non-human primate models demonstrated consistent immune responses across all sizes [150]. These findings underscore the complex relationship between LNPs size and immunogenic outcomes, suggesting species-specific nuances in vaccine responses [152]. Conclusively, the choice of LNP size is a critical factor in the effective delivery of mRNA constructs for CAR T therapy. While smaller LNPs demonstrate favorable characteristics for TME penetration, larger LNPs prove significant in eliciting immune responses. Additionally, the observed species-specific differences in immunogenicity emphasize the importance of considering these nuances in the development of mRNA-based therapies. Therefore, a systematic consideration of LNP size tailored to the therapeutic goals, target tissues, and species-specific characteristics is crucial for optimizing the delivery of mRNA constructs in CAR T therapy.

Extravasation may have implications for the effectiveness of LNP-mediated delivery of CAR-mRNA constructs in vivo. The pH-dependent binding kinetics of LNPs to the endosomal membrane before mRNA release is a critical factor [153]. The pH in extracellular environment can impact the binding and disintegration of LNPs, thereby influencing their delivery efficiency [154]. Additionally, the presence of a protein corona on the LNPs surface can impact their interaction with endosomal membranes [77]. The existence of lipoproteins in serum also plays a role in influencing LNPs uptake and mRNA-regulated protein production [155]. Moreover, the choice of ionizable lipid in the LNP formulation can have a significant effect on mRNA delivery efficacy [156].

In short, overcoming extracellular barriers in LNP-mediated mRNA delivery demands ongoing research. Strategies like PEG lipids incorporation for stability must be optimized for CAR construct delivery. Despite drawbacks, LNPs offer advanced protection against degradation, crucial for global vaccine distribution. Tailoring LNP size for specific immune responses is vital, emphasizing the need for further size-impact studies. Optimizing ionizable lipids enhances transfection and mRNA integrity. To address extravasation challenges, ongoing research in pH-dependent binding kinetics and protein corona influence is essential. Exploring tailored solutions for specific therapeutic contexts remains crucial for advancing mRNA-based therapies.

### Intracellular barriers

A significant challenge within the cytoplasm involves the inefficient release of mRNA from endosomes after cellular uptake. Only a small portion of external macromolecules can escape endosomes through mechanisms that are not fully understood. Previously, a significant research has shown improved endosomal escape post-modification of lipid components in LNPs, for instance, adding cationic lipids [157], incorporating DOPE, more content of ionic lipids and mRNA size ratios [117], and replacing the lipids with those which are highly expressed in vesicles and have promising role in modulation of intracellular signaling [158]; however, the delivery efficacy was compromised in these studies. Later, using cholesterol analogues added in LNPs design boosted the transfection of mRNA, and these polyhedral LNPs, with a slight difference in interior, showed increased uptake, prolonged retention, and perpetual endosomal escape [159].

Additionally, there are various other modifications for LNP optimization with promising results, for instance, including ionizable lipids in LNPs [160]. The one advantage of ionic lipids is that they stay neutral under normal pH range, however, they tend to reshape into cationic lipids when get exposed to lower pH, this property promotes binding to endosome membrane thereby destabilizing the LNPs and promoting escape [161]. One strategy could be the modification of nanostructure of LNPs that can influence endosomal escape efficiency without introducing additional components [66]. Introducing structurally active lipids enhances LNPs endosomal fusion, facilitating rapid evasion of endosomal entrapment and effective RNA delivery. For instance, RNA-LNPs with cuboplex nanostructures, while conserving lipid composition, exhibited significantly improved endosomal escape compared to traditional lipoplex constructs [162]. An investigational analysis using super-resolution microscopy revealed that various LNPs already used in mRNA vaccines acquire different capabilities for impairment of acidification in endosome, which causes the mRNAs to accumulate in sub-endosomal membranes unproductively [163]. This study provided more information on endosomal compartments that support mRNA escape. It revealed that Rab11 endosomes are more likely to facilitate mRNA escape compared to multivesicular bodies, late endosomes, lysosomes, and autophagosomes which are less likely to contribute to endosomal escape [163]. The endolysosomal pathway poses a significant intracellular barrier to the delivery of mRNA cargoes as these therapeutics commonly rely on endosomal uptake for cellular delivery [164]. Efforts to overcome these bottlenecks involve understanding the mechanisms of endosomal escape and identifying strategies to enhance the efficiency of mRNA escape from endosomes. Studies using live-cell imaging and galectin 8-GFP reporter systems have provided insights into the trafficking and escape capabilities of LNPs containing different chemical compositions, aiming to improve gene delivery efficacy [165]. Further research in this area is crucial to advance the field of nucleic acid therapies and optimize LNPs-mediated mRNA delivery for various applications, including cancer treatment.

In summary, there is a pressing need for a thorough mechanistic elucidation of endosomal escape, taking into account the intricacies that are essential for overcoming intracellular barriers to CAR-mRNA delivery. Focused research into novel modifications in LNPs goes beyond the conventional methods. By looking at different combinations of lipids and nano formulations, with an aim to enhance endosomal escape. Additionally, the optimization of ionizable lipids in LNPs, acknowledged for their role in promoting endosomal escape, necessitates research aimed at refining lipid formulations for maximal efficacy. Crucially, experts emphasize the importance of translating research findings into clinical applications, particularly in the field of cancer immunotherapy. This transition is seen as vital for optimizing LNPs-mediated mRNA delivery across various therapeutic applications. By addressing these specific research directions, the potential exists to propel the field of nucleic acid therapies forward and unlock novel avenues for effective intracellular delivery, particularly in the context of CAR constructs for cancer immunotherapy.

## Clinical considerations

LNPs have exhibited noteworthy potential in preclinical investigations for CAR T cell engineering, evolving as a prospective alternative to existing methodologies. While these LNPs for CAR T cells have not yet progressed to clinical trials, compelling preclinical results underscore their promise. A recent study, published in Molecular Therapy–Methods (2023), revealed that LNPs surpassed electroporation in the delivering mRNA during CAR T cell engineering. This underscores LNPs as a highly promising alternative that merits further exploration in clinical trials [28]. Moreover, several researchers have reported in vivo CAR T cell production using LNPs, thus offering a convenient and potentially safer avenue for CAR T cell therapy [79, 161]. These collective findings signify that LNPs for CAR T cell engineering, while still in the preclinical phase, hold substantial promise and merit consideration for future clinical translation. Thus, before implementing LNPs in clinical settings, several critical aspects should be considered to ensure their effectiveness and safety. These considerations include immunogenicity, the type of nucleic acid, reduced toxicity, improved safety, scalability, and clinical applicability. Recent research is making significant strides in addressing these considerations, propelling LNPs as a potential game-changer in CAR T cell therapy.

Significantly, the LNPs, which encapsulated Spy-Cas9 mRNA, T cell receptors, and CD52 guide RNA, were crafted through microfluidics, ensuring efficient transportation of the genetic cargo. While the successive addition of TCR and CARs demonstrated simultaneous CAR expression and TCR gene knockout, producing "off-the-shelf" CAR T cells that effectively cleared leukemia target cells [166]. This study underscores the leads of LNPs for the delivery of RNA to T cells, providing a gentle and versatile method coupled with microfluidics-based manufacturing, thereby enabling the efficient RNA libraries screening and the quick scaling-up of lead candidates. The transient expression of in situ CAR not only enhances the manageability of cytokine release but also addresses intricacies associated with tumor death, providing distinct advantages over conventional autologous cell therapy [167]. Sequential libraries of LNPs with wide-ranging excipient compositions were assessed against a typical formulation to augment the delivery of mRNA to T lymphocytes while reducing the cytotoxicity. Among the formulations tested, B10 emerged as the top performer, exhibiting a remarkable 3 times increase in efficiency of mRNA delivery. Comparatively, these LNPs demonstrated significant CAR expression levels as that in electroporation but with reduced cytotoxicity. Importantly, B10 LNPs exhibited potent cancer cell-killing capabilities [77].

One crucial aspect is the immunogenicity of LNPs which can be tailored to preferentially accumulate in specific organs, providing a level of control over their biodistribution. For example, specific LNPs formulations have shown a preference for liver delivery, emphasizing the versatility of LNPs in addressing clinical requirements [79]. The adaptable characteristics of LNPs make them a encouraging platform for CAR T cell engineering, with minimal concerns about immunogenicity.

The choice of nucleic acid is another critical consideration, and LNPs have shown efficacy in delivering nucleic acids, including mRNA and siRNA. Using iLNPs enables simultaneous therapeutic gene expression and RNA interference in T cells, showcasing the versatility of LNPs in manipulating cellular functions [82]. Understanding the intricacies of mRNA packaging within LNPs is vital for optimizing structure–property-function relationships in CAR loaded LNPs development. These characteristics make LNPs a valuable tool for mRNA-based CAR T cell therapy.

Reducing toxicity and improving safety is paramount for the clinical success of any therapeutic approach. LNPs have demonstrated less T cell toxicity compared to electroporation, indicating their potential to augment mRNA-based CAR T cell engineering techniques while maintaining safety standards. Additionally, LNPs offer a transient expression of CAR T cells, mitigating long-term off-target effects and toxicities associated with permanent CAR expression [63]. The transient nature of LNP-engineered CAR T cells aligns with broader trend of exploring non-integrative systems to improve the safety profiles in gene therapy [94]. In terms of scalability and clinical applicability, LNPs present several advantages. Their rapid optimization, absence of complex production requirements, and clinical applicability make them a viable option for large-scale CAR T cell engineering [81]. LNPs can be formulated with various lipid compositions, offering flexibility in engineering LNPs tailored for specific therapeutic applications [60, 110].

Clinically, evaluating LNPs in CAR T cell therapy is imperative to address challenges associated with the persistence and efficacy of CAR T cells. LNPs offer innovative solutions, such as in vivo studies with Pi-lipids, which identify specific lipid traits enhancing mRNA delivery. These insights provide valuable information for integrating LNPs into CAR T cell therapy [92]. Moreover, studies highlighted the promise of LNPs to improve ex vivo gene editing for CAR T cell therapy demonstrating improved clonogenic activity and comparable or superior reconstitution by long-term repopulating HSPCs, thus, emphasizing their potential in improving CAR T cell therapy [117].

In translation of CAR T cell engineering delivered by LNPs, there are several translational challenges which highlight the complexities in transitioning these technologies from preclinical to clinical settings. While preclinical studies have demonstrated the potential of LNPs in CAR T cell engineering, particularly, aforementioned approaches such as, delivering mRNA and enhancing CAR expression, their translation to clinical trials is yet to be realized [28, 53, 161]. Key considerations, including immunogenicity, nucleic acid selection, toxicity reduction, safety improvement, scalability, and clinical applicability, must be carefully addressed before clinical implementation [60, 63, 77, 79, 81, 82, 94, 110]. LNPs offer versatile platforms for mRNA-based CAR T cell therapy, with potential benefits such as transient expression, reduced off-target effects, and simplified production processes [63, 94]. Clinical evaluations are crucial for assessing the persistence and efficacy of LNP-mediated CAR T cell therapy, and identifying specific lipid traits that enhance mRNA delivery for improved therapeutic outcomes [92, 117]. Despite these translational challenges, ongoing research efforts continue to drive the optimization and integration of LNPs into CAR T cell therapy, holding promise for advancing the field towards safer and more effective treatments.

## Conclusion and future prospects

LNPs are credited with the advancement of cancer immunotherapy, specifically in CAR T cell therapy, marking a transition toward nonviral transduction approaches. Viral vectors have limitations in CAR engineering thus emphasizing the advantages of LNPs, including efficient gene delivery, reduced immunogenicity, and enhanced safety. Despite the potential benefits, it is crucial to comprehensively comprehend the adverse effects linked to LNP formulations to ensure the safe advancement of therapeutic interventions. Various mechanisms contribute to adverse responses, including IgE-mediated allergy, IgM-mediated pseudoallergy, and autoimmune reactions [91, 168]. Understanding these mechanisms is essential for mitigating potential adverse effects and developing strategies to enhance the safety profile of LNPs in therapeutic applications. Notwithstanding, there is a growing trend in research toward exploring LNPs as a promising and versatile nonviral delivery system, not only in CAR T cell therapy but also in other drug delivery applications. The potential of LNPs in various drug delivery routes is acknowledged, showcasing their versatility in the field. Moreover, a targeted exploration of novel modifications for LNPs entails surpassing established methodologies. This involves investigating distinctive lipid compositions and nanostructures to enhance endosomal escape efficiency. The objective should be to optimize the delivery system, facilitating efficient release from endosomes and thereby enhancing the overall therapeutic efficacy.

To sum up, LNPs exhibit significant potential in revolutionizing CAR T cell therapy, showcasing superior performance over traditional methods in preclinical studies. LNPs address critical considerations, including immunogenicity, nucleic acid type, reduced toxicity, safety, scalability, and clinical applicability. Recent advances highlight the versatility of LNPs in delivering various kinds of RNAs, enhancing transient CAR T cell generation with minimal toxicity. Promising preclinical results indicate LNPs as a prospective alternative, warranting exploration in clinical trials. As research advances, LNPs are positioned to herald a new era of CAR T cell therapies that are both safer and more effective, providing optimism for enhanced patient outcomes. Subsequent research efforts should concentrate on progressing clinical trials to substantiate these preclinical findings and fully unlock the potential of LNPs in the realm of CAR T cell therapy.

## Data Availability

No data was synthesized in this study.

## Abbreviations

APC: Antigen-presenting cells
ATC: Adoptive T cell
BCL: B-cell lymphoma
CAR: Chimeric antigen receptor
CART: Charge-altering releasable transporters
CR: Complete response
DOPE: 1,2-Dioleoyl-*sn*-glycero-3-phosphoethanolamine
DSPC: 1,2-Distearoyl-*sn*-glycero-3-phosphocholine
FDA: Food and Drug Administration
FIND: Fast identification of nanoparticle delivery
HSPC: Hematopoietic stem/progenitor cells
LNP: Lipid nanoparticles
MM: Multiple myeloma
NK: Natural killer
NMR: Nuclear magnetic resonance
SORT: Selective organ targeting
TCR: T cell receptors
TLR: Toll-like receptor
